# Breast Cancer Characteristics in the Population of Survivors Participating in the World Trade Center Environmental Health Center Program 2002–2019

**DOI:** 10.3390/ijerph18147555

**Published:** 2021-07-15

**Authors:** Alan A. Arslan, Yian Zhang, Nedim Durmus, Sultan Pehlivan, Adrienne Addessi, Freya Schnabel, Yongzhao Shao, Joan Reibman

**Affiliations:** 1 Department of Obstetrics and Gynecology, New York University Langone Health, New York, NY 10016, USA; 2 Department of Population Health, New York University Langone Health, New York, NY 10016, USA; Yian.Zhang@nyulangone.org (Y.Z.); Yongzhao.Shao@nyulangone.org (Y.S.); 3 NYU Perlmutter Comprehensive Cancer Center, New York University, New York, NY 10016, USA; Freya.Schnabel@nyulangone.org; 4 Department of Medicine, New York University Langone Health, New York, NY 10016, USA; Nedim.Durmus@nyulangone.org (N.D.); Sultan.Pehlivan@nyulangone.org (S.P.); addessia1@nychhc.org (A.A.); Joan.Reibman@nyulangone.org (J.R.)

**Keywords:** breast cancer, environmental exposure, exposure assessment, world trade center, 9/11

## Abstract

The destruction of World Trade Center on 11 September 2001 exposed local community members to a complex mixture of known carcinogens and potentially carcinogenic substances. To date, breast cancer has not been characterized in detail in the WTC-exposed civilian populations. The cancer characteristics of breast cancer patients were derived from the newly developed Pan-Cancer Database at the WTC Environmental Health Center (WTC EHC). We used the Surveillance, Epidemiology, and End Results (SEER) Program breast cancer data as a reference source. Between May 2002 and 31 December 2019, 2840 persons were diagnosed with any type of cancer at the WTC EHC, including 601 patients with a primary breast cancer diagnosis (592 women and 9 men). There was a higher proportion of grade 3 (poorly differentiated) tumors (34%) among the WTC EHC female breast cancers compared to that of the SEER-18 data (25%). Compared to that of the SEER data, female breast cancers in the WTC EHC had a lower proportion of luminal A (88% and 65%, respectively), higher proportion of luminal B (13% and 15%, respectively), and HER-2-enriched (5.5% and 7%, respectively) subtypes. These findings suggest considerable differences in the breast cancer characteristics and distribution of breast cancer intrinsic subtypes in the WTC-exposed civilian population compared to that of the general population. This is important because of the known effect of molecular subtypes on breast cancer prognosis.

## 1. Introduction

The destruction and collapse of the World Trade Center (WTC) towers and surrounding buildings on 11 September 2001 was an unprecedented disaster, with large groups of rescue workers, local workers, residents, and commuters intensely exposed to a complex mixture of known or suspected carcinogens leading to concerns about elevated cancer risk in exposed populations [1,2,3,4,5,6,7,8,9].

Breast cancer is the most common cancer in American women, except for nonmelanoma skin cancer [10]. Breast cancer also affects men, however, cohorts of first responders, which predominantly include men, had too few breast cancer cases to study in early assessment of cancer outcomes [7,11]. The WTC dust and fumes contained a complex mixture of known and suspected carcinogens that were implicated in breast cancer etiology, such as polychlorinated biphenyls (PCBs), perfluoroalkyl substances (PFAS), and fire retardant materials. More recently, Li et al. using data through 2011 from the World Trade Center Health Registry at the New York City Department of Health and Mental Hygiene (DOHMH) reported significant excess of breast cancer among WTC exposed female civilians not involved in rescue and recovery (standardized incidence ratio (SIR) = 1.34 [95% CI: 1.15–1.55]) and nonsignificant association for female rescue and recovery workers (SIR = 1.22 [95% CI: 0.90–1.61]) [8]. A recent paper by Shapiro et al. found no excess of breast cancers among general responder workers participating in the WTC Health Programs between 2003 and 2013 (SIR = 0.94 [95% CI: 0.69–1.24]) [9].

Civilian populations not involved in the rescue and recovery activities (local community members) remain understudied in terms of WTC exposures and related outcomes. These populations include those who were working in the WTC towers or in the many surrounding offices, schools, stores, and restaurants (local workers), residents of the surrounding buildings (residents), as well as workers involved in the cleanup of the surrounding area (cleanup workers). Over 36,000 local workers and over 57,000 residents south of Canal Street in lower Manhattan were estimated to have potential WTC dust and fume exposure [12]. For some community members, exposure to the dust and smoke persisted for weeks or months after the event, with many cleanup workers not wearing the proper respiratory protection, and many residents and workers returning to inadequately cleaned buildings [13].

To date, breast cancer among females was not characterized in detail in the WTC-exposed civilian populations. The objective of the current report is to provide descriptive characteristics of female breast cancer among the WTC-exposed civilian population enrolled in the New York University/Bellevue Hospital (NYU/Bellevue) WTC Environmental Health Center (WTC EHC).

## 2. Methods

### 2.1. Study Participants

#### World Trade Center Environmental Health Center

As part of the World Trade Center Health Program (WTCHP), created by the Centers for Disease Control (CDC) and the National Institute of Safety and Occupational Health (NIOSH), the Bellevue Hospital WTC EHC was, until recently, the only program that provided medical and mental health treatment for local community members and cleanup workers exposed to the WTC disaster [14,15].

Eligibility criteria for enrollment to the WTC Health Program included the following locations, activities, and time periods: presence in the NYC disaster area defined as the area of Manhattan south of Houston Street, and any block in Brooklyn wholly or partially contained within a 1.5 mile radius of the former WTC complex and (1) exposure to dust or dust cloud on 9 November 2001; (2) worked, lived, attended school, child care, or adult day care for at least 30 days between 9 November 2001 and 1 October 2002; (3) worked as a cleanup worker or performed maintenance with extensive exposure to WTC dust, but activities do not meet general responder criteria between 9 November 2002 and 1 October 2002.

The WTC EHC program started through joint efforts of the local community, organized labor, and the medical community [14]. This program was included in the James Zadroga 9/11 Health and Compensation Act l H.R. 847 in 2010. Conditions included in this program were deemed “certifiable illnesses” and included aerodigestive and mental health disorders. Cancer was added as a “certifiable” condition in 2012. Cancer cases who were diagnosed prior to 2012 were still eligible to be included in the WTC EHC program if they met the minimum latency period defined as a time between exposure to the WTC dust and the date of individual’s initial cancer diagnosis. The minimum latency period for solid tumors, including breast cancer, was determined by the WTC Program Guidelines to be four years [16]. The coverage and representativeness of the WTC EHC program is the same as for civilians in the rest of the WTC Health Program. The information on lifestyle characteristics (e.g., smoking status) was self-reported through detailed questionnaires at the time of enrollment to the WTC EHC program. We collected and reported the information on lifestyle characteristics as of 9/11/01. The weight, height, and body mass index (BMI) were measured as of time of WTC EHC enrollment.

In contrast to many of the responder programs, the WTC EHC is ethnically diverse and about half of its population are women. Community members self-refer into this program and under law inclusion requires the presence of a “certifiable condition” linked to the WTC disaster [15]. As of 31 December 2019, 11,048 individuals were enrolled in the WTC EHC program, with 2840 individuals diagnosed with cancer [17]. Of them, 592 women and 9 men were diagnosed with breast cancer.

### 2.2. Characterization of Breast Cancer

The cancer characteristics of breast cancer patients are derived from the newly developed WTC EHC Pan-Cancer Database (PCDB) that was created to capture information on all cancer types in the WTC EHC and is linked with the current WTC EHC clinical database [18]. We utilize REDCap as a secure Federal Information Security Modernization Act (FISMA) and Health Insurance Portability and Accountability Act (HIPAA)-compliant environment to support data capture and management for the WTC EHC PCDB. The three main domains of the PCDB include patient demographics, site-specific cancer characteristics, and cancer biomarker information [18].

We acquired information on breast cancer characteristics from the WTC EHC PCDB, which includes data extracted from pathology reports, clinical records, and other available medical records. All available cancer data are carefully reviewed by a pathologist and clinicians at the WTC EHC. Breast cancer characteristics such as age at diagnosis, anatomic location of tumor (ICD-10 classification), tumor size, grade, histology (ICD-O code), TNM (Tumor size, Node involvement, Metastases status) classification, and disease stage are recorded for each breast cancer case.

We included in the current analysis all subjects with ductal carcinomas (ICD-O codes: 8140, 8500, 8501, 8502, 8503, 8504, 8505, 8514), lobular carcinomas (ICD-O code: 8520), and mixed carcinomas (ICD-O codes: 8521, 8522, 8523, 8524).

In addition to histological subtypes, several distinct molecular subtypes of breast cancer were defined based on gene expression profiles including luminal A, luminal B, HER2-enriched, normal-like, and triple-negative (basal-like and claudin-low) breast cancer subtypes [19,20,21]. The four major molecular subtypes of breast cancer can be approximated by the combined expression of three biomarkers: estrogen receptor (ER), progesterone receptor (PR), and human epidermal growth factor receptor 2-neu (HER2) that are routinely evaluated in breast cancer patients to guide clinical care. Recent findings suggest that immunohistochemical receptor expression profiles in breast tissues can serve as surrogates for gene-derived intrinsic subtypes [22,23]. We used immunohistochemical protein expression data for ER, PR, and HER2 from pathology reports to define four common molecular subtypes of breast cancer that can be approximated using receptor status: (1) luminal A (hormone receptor (ER and/or PR) positive, HER2-negative); (2) luminal B (hormone receptor (ER and/or PR) positive, HER2-positive); (3) triple-negative (hormone receptor (ER and PR) negative, HER2-negative); and (4) HER2-enriched (hormone receptor (ER and PR) negative, HER2-positive).

Second primary tumors, defined according the International Association of Cancer Registries and International Agency for Research on Cancer criteria were also recorded in the WTC EHC PCDB. Notably, medical facilities where the biopsies and surgeries were performed are included in the WTC EHC PCDB to enable future procurement of tumor tissue samples.

We used the Surveillance, Epidemiology, and End Results (SEER) Program breast cancer data as a reference source. The SEER-18 covers the period from 2000 through 2018. The SEER-21 covers the same period but also includes the data on hormone receptors (ER, PR, HER2) and molecular classification of breast cancer.

Cancer-free subjects among WTC-exposed included subjects with respiratory and digestive disorders. The cancer-free subjects are from the same source population and share similar risk factors as breast cancer patients.

All subjects were asked to provide informed consent before they entered the WTC EHC Program. The study was conducted in accordance with the Declaration of Helsinki, and the protocol was approved by the New York University School of Medicine Institutional Review Board (IRB number: i06-1). Patients with breast cancer were analyzed using deidentified dataset after removal of personal identifiers (IRB approval number: i06-1_MOD49).

### 2.3. Data Analysis

WTC exposure, demographic, and tumor characteristics of breast cancer patients were summarized by descriptive statistics, including medians and interquartile ranges for continuous variables, and counts and percentages for dichotomous or categorical variables. To compare the difference between specific groups (e.g., WTC exposure groups) for certain characteristics (e.g., molecular subtypes of breast cancer), we utilized chi-square test or Fisher’s exact test for categorical variables and Mann–Whitney test for continuous variables. Significance level is set to 0.05 for the two-sided tests. Data were analyzed using statistical software *R* (version *R*-3.6.3, R Foundation, Wien, Austria).

## 3. Results

Between May 2002 and 31 December 2019, 11,038 individuals enrolled in the WTC EHC Program. Among them, 2840 persons were diagnosed with any type of cancer, including 601 patients with a primary breast cancer diagnosis (592 women, 9 men). Breast cancer is the most frequent cancer among women in the WTC EHC, accounting for 46% of all female cancer diagnoses [17]. Among 592 female patients with breast cancer, 41 women had two primary breast cancer diagnoses resulting in a total of 633 (512 invasive and 121 in situ) female breast cancer diagnoses.

Table 1 provides descriptive characteristics of female breast cancer patients (*n* = 592) and female non-cancer participants (*n* = 2683) of the WTC EHC who signed informed consent. Compared to that of cancer-free subjects, breast cancer patients had a higher median age (42.5 vs. 43.5 years, respectively, *p* = 0.01) at the time of initial WTC exposure on 9 November 2001. The WTC EHC population is racially and ethnically diverse, including about 30% of Hispanic women, 27% of non-Hispanic black, 10% of non-Hispanic Asian, and 33% of non-Hispanic white women. Relative to the cancer-free WTC EHC female population, breast cancer cases had lower proportion of Hispanic women (30% and 14%, respectively) and higher proportion of non-Hispanic Asian women (10% and 16%, respectively). There was a higher proportion of women with higher income and education level beyond high school among female breast cancer patients compared to noncancer patients in the WTC EHC (as illustrated in Table 1). Distribution of smoking status and pack-years of smoking were similar between female breast cancer patients and noncancer patients (as illustrated in Table 1). Descriptive characteristics of 9 male breast cancer patients are presented in Supplemental Table S1.

Table 2 describes tumor characteristics of primary breast cancers diagnosed among females in the WTC EHC in comparison to the Surveillance, Epidemiology, and End Results (SEER-18) Program data [24]. The most common histological subtypes of breast cancers in the WTC EHC were invasive ductal carcinoma (82%), followed by invasive lobular carcinoma (9%); rates that are similar to the SEER-18 data. There was a higher proportion of grade 3 (poorly differentiated) tumors (34%) among the WTC EHC female breast cancers compared to that of the SEER-18 data (25%). Compared to that of the SEER-18 data, female breast cancer cases in the WTC EHC had lower proportion of large size (≥5 cm) primary tumors (9% and 4%, respectively), lower positivity of regional lymph nodes (28% and 20%, respectively), and lower proportion of tumors with distant metastases (stage IV) (6% and 2%, respectively). Tumor characteristics of male breast cancer cases are presented in Supplemental Table S2.

Distribution of molecular subtypes of breast cancer in the WTC EHC in comparison to the corresponding available data from SEER Program is shown in Table 3. Compared to that of the SEER-21 (2014–2018) data, breast cancers in the WTC EHC had higher proportion of luminal B (10% and 15%, respectively), triple-negative (10% and 13%, respectively), and HER-2-enriched (4% and 7%, respectively) subtypes (as illustrated in Table 3).

Because molecular subtypes of breast cancer differ by race and ethnicity, Table 4 shows distribution of molecular subtypes of breast cancer by race/ethnicity in comparison to that of the available SEER-18 data [24]. Compared to the SEER-18 data, there was a higher proportion of triple-negative molecular subtype among Hispanic women at the WTC EHC (15% and 23%, respectively). Relative to the SEER-18 data, non-Hispanic black women at the WTC EHC had higher proportion of luminal B (11% and 17%, respectively) and lower proportion of luminal A (60% and 52%, respectively). Compared to that of the SEER-18 data, non-Hispanic Asian women at the WTC EHC had higher proportion of luminal A subtype (71% and 81%, respectively) and lower proportion of HER2-enriched subtype (7% and 2%, respectively).

Seventeen percent of women with breast cancer diagnosis at the WTC EHC (101 out of 592) had more than one primary cancer diagnosis. Eighty-three women (14%) had two primary cancer diagnoses, 14 women (2%) had 3 primary cancer diagnoses, and 5 women (1%) had more than three primary cancer diagnoses. Among 86 cases with nonbreast cancer second primary cancer diagnosis, 38 (44%) were diagnosed before and 48 (56%) were diagnosed after the breast cancer diagnosis. Table 5 shows the distribution of second primary cancers among female breast cancer cases at the WTC EHC as of 12/31/2019. Among second primary cancer diagnoses, the most common were another primary breast cancer (31%) and nonmelanoma skin cancer (25%) among solid cancers and lymphoma (4%) and leukemia (3%) among blood cancers (as illustrated in Table 5).

## 4. Discussion

Breast cancer is the most common cancer among women enrolled in the WTC EHC. The WTC EHC is racially and ethnically diverse, and women comprise about half of the WTC EHC population. Relative to the cancer-free WTC EHC female population, we observed lower proportion of Hispanic women and higher proportion of non-Hispanic Asian women among breast cancer patients. This could be a reflection of self-referral of patients to the WTC EHC and could also reflect the well-established impact of socio-economic status on breast cancer [26,27,28,29,30,31,32,33]. Using proxy markers of socio-economic status, such as individual income and degree of education, we observed an increased proportion of women with higher income and education level beyond high school among female breast cancer patients compared to that of noncancer patients in the WTC EHC program.

We also observed a higher proportion of grade 3 (poorly differentiated) tumors among the WTC-exposed female breast cancer patients compared to the SEER-18 data. Poorly differentiated breast cancer tumors are characterized by specific set of over-expressed genes (e.g., UBE2C, CCNB2, CDK1, KIF2C, NDC80, and CCNB2) and are associated with more aggressive tumors and lower survival [34]. This finding is concerning and needs to be confirmed by independent studies.

Relative to the SEER-18 data, female breast cancer cases in the WTC EHC had lower proportion of large size (≥5 cm) primary tumors, lower positivity of regional lymph nodes, and lower proportion of tumors with distant metastases (stage IV) at the time of diagnosis. This could be a reflection of screening and early detection of breast cancer in the WTC EHC patients.

We used immunohistochemical protein expression data for ER, PR, and HER2 from pathology reports to describe four common molecular subtypes of breast cancer, as previously described [22,23]. Luminal B breast cancers and two other subtypes, HER2-enriched and triple-negative tumors (most of which are basal-like tumors), are known to be more clinically aggressive and have poorer prognosis compared to luminal A tumors [24,35,36]. Compared to that of the SEER-18 data, we observed a higher proportion of triple-negative tumors among Hispanic women at the WTC EHC. Relative to the SEER-18 data, non-Hispanic black women at the WTC EHC had higher proportion of luminal B tumors and lower proportion of luminal A tumors. Compared to that of the SEER-18 data, non-Hispanic Asian women at the WTC EHC had higher proportion of luminal A tumors and lower proportion of HER2-enriched tumors. These findings suggest substantial differences in the distribution of breast cancer intrinsic subtypes among racial and ethnic groups in the WTC EHC program. This is important because of the known effect of molecular subtypes on breast cancer survival and prognosis with luminal A breast tumors characterized by the highest survival and triple-negative breast tumors associated with the lowest survival [37].

Substantial proportion (17%) of the breast cancer patients in the WTC EHC program had more than one primary cancer diagnosis. Among second primary cancer diagnoses, the most common were second primary breast cancer and nonmelanoma skin cancer among solid cancers and lymphoma and leukemia among blood cancers. Multiple primary cancers could reflect the effects of gene-environment interactions among WTC-exposed patients and should be subject of future studies.

The study has several limitations. Because the study is based on a self-referred population, we were unable to estimate incidence of breast cancer in the WTC EHC program. Self-referral of the patients also raises the possibility of selection bias for patients who lack health insurance or have inadequate insurance status since the WTC EHC program covers patients who are certified as exposed to the WTC disaster. Potential screening and early detection bias could explain lower than expected proportion of advanced and metastatic breast cancers in the WTC EHC compared to that of the SEER-18 data. Lack of BRCA1/2 status and Ki-67 data for many breast cancer patients is another limitation of the current study. The lower proportion of later stage breast cancer cases in the WTC EHC may also be a result of survivor bias, i.e., those with more aggressive disease possibly died prior to the time of program enrollment and were not included. The sample size in some subset analyses by race/ethnicity is small and caution is required while interpreting the results. In addition, we did not have data on several important risk factors for breast cancer, such as physical activity, menopausal status, and reproductive history for these patients.

Despite these limitations, the study is the first comprehensive description of female breast cancer in a WTC-exposed civilian population to date. Inclusion of women and minorities is a strength of the current study compared to most of previous studies based on the first responders. This is the first study providing molecular classification of female breast cancer using biomarker data in the WTC-exposed population.

Future research should address the potential racial and ethnic differences in molecular subtypes of breast cancer observed in the current study. Studies utilizing tissue samples and analyzing functional pathways dysregulated among the WTC-exposed cancer patients and patients with multiple primary cancers would be particularly informative.

## Figures and Tables

**Table 1:** Demographic characteristics of female patients in the WTC EHC with and without breast cancers as of 31 December 2019.

Characteristic		Non Cancer *	Breast Cancer	*p*-Value
		2683	592	
Age on 9/11, median [interquartile range]		42.5 (34.6, 49.7)	43.5 (36.6, 50.5)	0.01
Age at diagnosis, median [interquartile range]		-	54.0 (48.0, 62.8)	-
Age group on 9/11, *n* (%)	<20	162 (6)	4 (1)	<0.001
	21–29	258 (10)	52 (9)	
	30–39	683 (25)	165 (28)	
	40–49	920 (34)	209 (35)	
	50–59	532 (20)	140 (24)	
	60–69	98 (4)	19 (3)	
	70–79	28 (1)	3 (1)	
	≥80	2 (0)	0 (0)	
Race/Ethnicity, *n* (%)	Hispanic	784 (30)	66 (14)	<0.001
	Non-Hispanic white	867 (33)	204 (42)	
	Non-Hispanic black	699 (27)	133 (28)	
	Non-Hispanic Asian	260 (10)	78 (16)	
	Native American	9 (0)	0 (0)	
BMI, *n* (%)	Normal weight (<25)	805 (34)	131 (35)	0.61
	Overweight (25–30)	726 (30)	116 (31)	
	Obese (≥30)	856 (36)	123 (33)	
Income, *n* (%)	≤$30,000/year	1372 (53)	191 (40)	<0.001
	>$30,000/year	1195 (47)	281 (60)	
Education, *n* (%)	High school or less	809 (30)	123 (25)	0.03
	More than high school	1873 (70)	366 (75)	
Smoking status, *n* (%)	Never (≤1 p-y)	1887 (72)	356 (73)	0.43
	Former and current smokers (>1 p-y, stopped)	751 (28)	129 (27)	
Smoking pack-years, *n* (%)	≤5 pack-year	2142 (81)	392 (81)	0.90
	>5 pack-year	496 (19)	93 (19)	

* Includes only those with signed consent, all % rounded.

**Table 2:** Breast cancer characteristics among female patients in WTC EHC as of 31 December 2019.

Characteristic		WTC EHC *n* (%)	SEER-18 [24] *n* (%)
		*n* = 592	*n* = 57,483
Laterality, *n* (%)	Left	288 (49)	
	Right	297 (50)	
	Bilateral	5 (1)	
	Unknown	2 (0)	
Grade, *n* (%)	G1. Well-differentiated	81 (14)	13,158 (23)
	G2. Moderately differentiated	239 (40)	20,562 (36)
	G3. Poorly differentiated	194 (33)	14,157 (25)
	Unknown	78 (13)	9606 (17)
Histology, *n* (%)	Ductal	494 (83)	
	Lobular	49 (8)	
	Mixed	23 (4)	
	Unknown	26 (4)	
pT (Primary tumor), *n* (%)	T1 (<2.0 cm)	314 (65)	30,763 (54)
	T2 (2.0–4.9 cm)	105 (22)	18,614 (32)
	T3-T4 (≥5.0 cm)	19 (4)	5036 (9)
	Unknown	44 (9)	3070 (5)
pN-Regional lymph nodes, *n* (%)	N0. No regional lymph node metastasis	333 (56)	32,891 (57)
	N1. Regional lymph node metastasis	117 (20)	16,085 (28)
	Unknown	142 (24)	8507 (15)
pM-Distant metastasis, *n* (%)	M0. No distant metastasis	541 (91)	51,815 (90)
	M1. Distant metastasis	10 (2)	3203 (6)
	Unknown	41 (7)	2390 (4)
Stage, AJCC * (%)	I	273 (57)	27,816 (48)
	II	127 (26)	17,494 (30)
	III	32 (7)	6505 (11)
	IV	10 (2)	3203 (6)
	Unknown	41 (8)	2390 (4)

* Because available SEER data did not report in situ breast cancer cases, Table 2 did not include 121 in situ breast cancer diagnoses in the WTC EHC for comparison purposes.

**Table 3:** Molecular classification of breast cancer subtypes among female breast cancer patients in WTC EHC in comparison to that of SEER-21 data [25].

Breast Cancer Subtype	Hormone Receptor Status	HER2 Status	WTC EHC (2002–2019) *n* (%)	SEER 21 (2014–2018) (%)
Luminal A (HR+/HER2-)	HR+ (ER+ and/or PR+)	-	266 (64)	88.1
Luminal B (HR+/HER2+)	HR+ (ER+ and/or PR+)	+	65 (16)	13.4
Triple-negative (HR-/HER2-)	HR- (ER- and PR-)	-	56 (13)	13.1
HER2-enriched (HR-/HER2+)	HR- (ER- and PR-)	+	29 (7)	5.5

Note: all three receptors’ (ER, PR, HER2) statuses were available for 432 (68%) of female breast cancer diagnoses. The three receptors’ statuses were incomplete or unknown for 201 (32%) of female breast cancer diagnoses in the WTC EHC.

**Table 4:** Breast cancer subtypes by race/ethnicity in WTC EHC in comparison to that of SEER-18 data [24].

Breast Cancer Subtype	Race/Ethnicity							
	NH White		NH Black		NH Asian		Hispanic	
	WTC EHC %	SEER %	WTC EHC %	SEER %	WTC EHC %	SEER %	WTC EHC %	SEER %
Luminal A (HR+/HER2-), *n* (%)	105 (69)	76	47 (52)	60	39 (81)	71	26 (55)	68
Luminal B (HR+/HER2+), *n* (%)	24 (16)	10	15 (17)	11	4 (8)	12	8 (17)	11
Triple-negative (HR-/HER2-), *n* (%)	14 (9)	11	21 (23)	22	4 (8)	10	11 (23)	15
HER2-enriched (HR-/Her2+), *n* (%)	10 (7)	4	7 (8)	6	1 (2)	7	2 (4)	6

**Table 5:** Distribution of female breast cancers with more than one primary cancer diagnosis at WTC EHC as of 31 December 2019.

Second Primary Cancer Type	*n* (%)
Breast	43 (33)
Carcinoma of skin, non-melanoma	32 (25)
Thyroid	8 (6)
Lung	7 (5)
Colon and rectum	6 (4)
Melanoma	5 (4)
Kidney	3 (2)
Ovary	3 (3)
Corpus uteri	2 (2)
Pancreas	1 (1)
Small intestine	1 (1)
Urinary bladder	1 (1)
Vagina	1 (1)
Vulva	1 (1)
Ophtalmic	1 (1)
Esophagus	1 (1)
Head and neck	1 (1)
Subtotal Solid Cancers	117 (91)
Lymphoma	5 (4)
Leukemia	4 (3)
Myeloma	3 (2)
Subtotal Blood Cancers	12 (9)
Total	129 (100)

## Data Availability

The data that support the findings of this study are available on request from the corresponding author. The data are not publicly available due to restrictions, such as containing information that could compromise the privacy of research participants.

## Supplementary Materials

The following are available online at https://www.mdpi.com/article/10.3390/ijerph18147555/s1, Table S1: Demographic characteristics of male patients in the WTC EHC with and without breast cancers as of 31 December 2019, Table S2: Breast cancer characteristics among male patients in the WTC EHC as of 31 December 2019, Table S3: Age-adjusted odds ratios (and 95% CIs) comparing female breast cancer cases vs. non-cancer controls using multiple logistic regression.

## References

[1] Lioy P.J., Weisel C.P., Millette J.R., Eisenreich S., Vallero D., Offenberg J., Buckley B., Turpin B., Zhong M., Cohen M.D. (2002). Characterization of the dust/smoke aerosol that settled east of the World Trade Center (WTC) in lower Manhattan after the collapse of the WTC 11 September 2001. Environ. Health Perspect..

[2] McGee J.K., Chen L.C., Cohen M.D., Chee G.R., Prophete C.M., Haykal-Coates N., Wasson S.J., Conner T.L., Costa D.L., Gavett S.H. (2003). Chemical analysis of World Trade Center fine particulate matter for use in toxicologic assessment. Environ. Health Perspect..

[3] Landrigan P.J., Lioy P.J., Thurston G., Berkowitz G., Chen L.C., Chillrud S.N., Gavett S.H., Georgopoulos P.G., Geyh A.S., Levin S. (2004). Health and environmental consequences of the world trade center disaster. Environ. Health Perspect..

[4] Solan S., Wallenstein S., Shapiro M., Teitelbaum S.L., Stevenson L., Kochman A., Kaplan J., Dellenbaugh C., Kahn A., Biro F.N. (2013). Cancer Incidence in World Trade Center Rescue and Recovery Workers, 2001–2008. Environ. Health Perspect..

[5] Kleinman E.J., Christos P.J., Gerber L.M., Reilly J.P., Moran W.F., Einstein A.J., Neugut A.I. (2015). NYPD Cancer Incidence Rates 1995–2014 Encompassing the Entire World Trade Center Cohort. J. Occup. Environ. Med..

[6] Boffetta P., Zeig-Owens R., Wallenstein S., Li J., Brackbill R., Cone J., Farfel M., Holden W., Lucchini R., Webber M.P. (2016). Cancer in World Trade Center responders: Findings from multiple cohorts and options for future study. Am. J. Ind. Med..

[7] Moir W., Zeig-Owens R., Daniels R.D., Hall C.B., Webber M.P., Jaber N., Yiin J.H., Ms T.S., Liu X., Ms M.V. (2016). Post-9/11 cancer incidence in World Trade Center-exposed New York City firefighters as compared to a pooled cohort of firefighters from San Francisco, Chicago and Philadelphia (9/11/2001–2009). Am. J. Ind. Med..

[8] Li J., Brackbill R.M., Liao T.S., Qiao B., Cone J.E., Farfel M.R., Hadler J.L., Kahn A.R., Konty K.J., Stayner L.T. (2016). Ten-year cancer incidence in rescue/recovery workers and civilians exposed to the September 11, 2001 terrorist attacks on the World Trade Center. Am. J. Ind. Med..

[9] Shapiro M.Z., Wallenstein S.R., Dasaro C.R., Lucchini R.G., Sacks H.S., Teitelbaum S.L., Thanik E.S., Crane M.A., Harrison D.J., Luft B.J. (2020). Cancer in General Responders Participating in World Trade Center Health Programs, 2003–2013. JNCI Cancer Spectr..

[10] American Cancer Society (2021). Cancer Facts & Figures 2021.

[11] Zeig-Owens R., Webber M.P., Hall C.B., Schwartz T., Jaber N., Weakley J., Rohan T.E., Cohen H.W., Derman O., Aldrich T.K. (2011). Early assessment of cancer outcomes in New York City firefighters after the 9/11 attacks: An observational cohort study. Lancet.

[12] Brackbill R.M., Thorpe L.E., DiGrande L., Perrin M., Sapp J.H., Wu D., Campolucci S., Walker D.J., Cone J., Pulliam P. (2006). Surveillance for World Trade Center disaster health effects among survivors of collapsed and damaged buildings. Morb. Mortal. Wkly. Rep. Surveill. Summ..

[13] Lioy P.J., Georgopoulos P. (2006). The Anatomy of the Exposures That Occurred around the World Trade Center Site: 9/11 and Beyond. Ann. N. Y. Acad. Sci..

[14] Reibman J., Liu M., Cheng Q., Liautaud S., Rogers L., Lau S., Berger K., Goldring R., Marmor M., Fernandez-Beros M.E. (2009). Characteristics of a Residential and Working Community With Diverse Exposure to World Trade Center Dust, Gas, and Fumes. J. Occup. Environ. Med..

[15] Reibman J., Levy-Carrick N., Miles T., Flynn K., Hughes C., Crane M., Lucchini R. (2016). Destruction of the World Trade Center Towers. Lessons Learned from an Environmental Health Disaster. Ann. Am. Thorac. Soc..

[16] Howard J. (2015). Minimum Latency & Types or Categories of Cancer. World Trade Center Health Program. https://www.cdc.gov/wtc/pdfs/policies/WTCHP-Minimum-Cancer-Latency-PP-01062015-508.pdf.

[17] Durmus N., Shao Y., Arslan A.A., Zhang Y., Pehlivan S., Fernandez-Beros M.E., Umana L., Corona R., Smyth-Giambanco S., Abbott S.A. (2020). Characteristics of Cancer Patients in the World Trade Center Environmental Health Center. Int. J. Environ. Res. Public Health.

[18] Shao Y., Durmus N., Zhang Y., Pehlivan S., Fernandez-Beros M.E., Umana L., Corona R., Abbott S., Smyth-Giambanco S., Arslan A.A. (2021). The Development of a WTC Environmental Health Center Pan-Cancer Database. Int. J. Environ. Res. Public Health.

[19] Perou C.M., Sørlie T., Eisen M.B., Van De Rijn M., Jeffrey S.S., Rees C.A., Pollack J.R., Ross D.T., Johnsen H., Akslen L.A. (2000). Molecular portraits of human breast tumours. Nature.

[20] Sorlie T., Perou C.M., Tibshirani R., Aas T., Geisler S., Johnsen H., Hastie T., Eisen M.B., van de Rijn M., Jeffrey S.S. (2001). Gene expression patterns of breast carcinomas distinguish tumor subclasses with clinical implications. Proc. Natl. Acad. Sci. USA.

[21] Sørlie T., Tibshirani R., Parker J., Hastie T., Marron J.S., Nobel A., Deng S., Johnsen H., Pesich R., Geisler S. (2003). Repeated observation of breast tumor subtypes in independent gene expression data sets. Proc. Natl. Acad. Sci. USA.

[22] Prat A., Cheang M.C.U., Martín M., Parker J.S., Carrasco E., Caballero R., Tyldesley S., Gelmon K., Bernard P.S., Nielsen T.O. (2013). Prognostic Significance of Progesterone Receptor–Positive Tumor Cells within Immunohistochemically Defined Luminal A Breast Cancer. J. Clin. Oncol..

[23] Prat A., Pineda E., Adamo B., Galván P., Fernandez-Martinez A., Gaba L., Díez M., Viladot M., Arance A., Munoz M. (2015). Clinical implications of the intrinsic molecular subtypes of breast cancer. Breast.

[24] Howlader N., Altekruse S.F., Li C.I., Chen V.W., Clarke C.A., Ries L.A., Cronin K.A. (2014). US Incidence of Breast Cancer Subtypes Defined by Joint Hormone Receptor and HER2 Status. J. Natl. Cancer Inst..

[25] SEER* Stat Database: Cancer Stat Facts: Female Cancer Subtypes, SEER 21 (2014–2018), National Cancer Institute, DCCPS, Surveillance Research Program. www.seer.cancer.gov/statfacts/html/breast-subtypes.html.

[26] Danø H., Hansen K., Jensen P., Petersen J.H., Lindahl-Jacobsen R., Ewertz M., Lynge E. (2004). Fertility pattern does not explain social gradient in breast cancer in denmark. Int. J. Cancer.

[27] Braaten T., Weiderpass E., Kumle M., Lund E. (2005). Explaining the Socioeconomic Variation in Cancer Risk in the Norwegian Women and Cancer Study. Cancer Epidemiol. Biomark. Prev..

[28] Hussain S.K., Altieri A., Sundquist J., Hemminki K. (2008). Influence of education level on breast cancer risk and survival in Sweden between 1990 and 2004. Int. J. Cancer.

[29] Larsen S.B., Olsen A., Lynch J., Christensen J., Overvad K., Tjønneland A., Johansen C., Dalton S.O. (2011). Socioeconomic position and lifestyle in relation to breast cancer incidence among postmenopausal women: A prospective cohort study, Denmark, 1993–2006. Cancer Epidemiol..

[30] Menvielle G., Kunst A.E., Van Gils C.H., Peeters P.H.M., Boshuizen H., Overvad K., Olsen A., Tjonneland A., Hermann S., Kaaks R. (2010). The Contribution of Risk Factors to the Higher Incidence of Invasive and In Situ Breast Cancers in Women with Higher Levels of Education in the European Prospective Investigation Into Cancer and Nutrition. Am. J. Epidemiol..

[31] Pudrovska T., Anikputa B. (2012). The Role of Early-Life Socioeconomic Status in Breast Cancer Incidence and Mortality. J. Aging Health.

[32] Akinyemiju T., Sakhuja S., Vin-Raviv N. (2016). Racial and socio-economic disparities in breast cancer hospitalization outcomes by insurance status. Cancer Epidemiol..

[33] Berger E., Maitre N., Mancini F.R., Baglietto L., Perduca V., Colineaux H., Sieri S., Panico S., Sacerdote C., Tumino R. (2020). The impact of lifecourse socio-economic position and individual social mobility on breast cancer risk. BMC Cancer.

[34] Jayanthi V.S.A., Das A.B., Saxena U. (2020). Grade-specific diagnostic and prognostic biomarkers in breast cancer. Genomics.

[35] Fan C., Oh D.S., Wessels L., Weigelt B., Nuyten D.S., Nobel A.B., Veer L.J.V., Perou C.M. (2006). Concordance among Gene-Expression–Based Predictors for Breast Cancer. N. Engl. J. Med..

[36] Millikan R.C., Newman B.M., Tse C.-K., Moorman P.G., Conway K., Smith L.V., Labbok M., Geradts J., Bensen J.T., Jackson S. (2007). Epidemiology of basal-like breast cancer. Breast Cancer Res. Treat..

[37] Howlader N., Cronin K.A., Kurian A.W., Andridge R. (2018). Differences in Breast Cancer Survival by Molecular Subtypes in the United States. Cancer Epidemiol. Biomark. Prev..

