# Supplementary material for: Breast Cancer Characteristics in the Population of Survivors Participating in the World Trade Center Environmental Health Center Program 2002–2019
Source: Int J Environ Res Public Health. 2021 Jul 15;18(14):7555. doi: 10.3390/ijerph18147555 (PMC8306152; doi:10.3390/ijerph18147555)
Supplement: Supplementary file 1 [file ijerph-18-07555-s001.zip › ijerph-1224959-supplementary.pdf]

International Journal of
Environmental Research
and Public Health

MDPI

Supplementary Materials

Table S1. Demographic characteristics of male patients in the WTC EHC with and without breast cancers as of 31 December 2019.

Characteristic		Non-Cancer *	Male Breast Cancer Patients	*p*-value
		2704	9	
Age on 9/11, median (interquartile range)		40.50 (32.60, 48.20)	51.60 (45.30, 54.10)	0.03
Age group on 9/11, *n* (%)	<20	157 (6)	0 (0)	0.03
	21–29	355 (13)	1 (11)	
	30–39	771 (29)	1 (11)	
	40–49	861 (32)	2 (22)	
	50–59	431 (16)	3 (33)	
	60–69	104 (4)	1 (11)	
	70–79	22 (1)	1 (11)	
	≥80	3 (0)	0 (0)	
Race/Ethnicity, *n* (%)	Hispanic	811 (31)	0 (0)	0.08
	Non-Hispanic white	1129 (43)	8 (89)	
	Non-Hispanic black	353 (13)	0 (0)	
	Non-Hispanic Asian	352 (13)	1 (11)	
	Native American	5 (0)	0 (0)	
BMI (Body Mass Index), *n* (%)	Normal weight (<25)	561 (24)	2 (25)	0.97
	Overweight (25–30)	991 (42)	3 (38)	
	Obese (≥30)	815 (34)	3 (38)	
Income, *n* (%)	≤$30,000/year	1470 (56)	2 (22)	0.09
	>$30,000/year	1146 (44)	7 (78)	
Education, *n* (%)	High school or less	1002 (37)	1 (11)	0.21
	More than high school	1699 (63)	8 (89)	
Smoking status, *n* (%)	Never (≤1 pack-year)	1784 (67)	6 (67)	0.99
	Former and current smokers (>1 pack-year, stopped)	880 (33)	3 (33)	
Smoking pack-years, *n* (%)	≤5 pack-year	2035 (76)	7 (78)	0.99
	>5 pack-year	629 (24)	2 (22)	

* Includes only those with signed consent, all % rounded. WTC EHC—World Trade Center Environmental Health Center.

Table S2. Breast cancer characteristics among male patients in the WTC EHC as of 31 December 2019.

Characteristic		Male Breast Cancer Diagnoses *n* (%)
		n = 11 *
Laterality, *n* (%)	Left	6 (55)
	Right	5 (45)
Grade, *n* (%)	G1. Well-differentiated	3 (27)
	G2. Moderately differentiated	5 (45)
	G3. Poorly differentiated	3 (27)
	Ductal	8 (73)
Histology, *n* (%)	Ductal in situ	3 (27)
pT (Primary tumor), *n* (%)	T0 (in situ)	3 (27)
	T1 (<2.0 cm)	6 (55)
	T2 (2.0–4.9 cm)	2 (18)
	T3-T4 (≥5.0 cm)	0 (0)
pN-Regional lymph nodes, *n* (%)	N0. No regional lymph node metastasis	7 (64)
	N1. Regional lymph node metastasis	2 (18)
	Unknown	2 (18)
pM-Distant metastasis, *n* (%)	M0. No distant metastasis	9 (82)
	M1. Distant metastasis	0 (0)
	Unknown	2 (18)
Stage, AJCC ** (%)	0	3 (27)

	I	5 (45)
	II	2 (18)
	III	1 (9)
	IV	0 (0)

* Includes 2 additional second primary breast cancer diagnoses among 2 male patients with two primary breast cancer diagnoses. ** Because available SEER data did not report in situ breast cancer cases, Table 2 did not include 121 in situ breast cancer diagnoses in the WTC EHC for comparison purposes. WTC EHC—World Trade Center Environmental Health Center.

Table S3. Age-adjusted odds ratios (and 95% CIs) comparing female breast cancer cases vs. non-cancer controls using multiple logistic regression.

**Race/ethnicity (Hispanic as reference group)**	**Odds Ratios**	**95% Confidence Interval**
Non-Hispanic White	2.74	(2.05, 3.70)
Non-Hispanic Black	2.18	(1.60, 2.99)
Asian or Native American	3.43	(2.40, 4.90)
Income >$30,000 /year vs. ≤$30,000/year	1.74	(1.42, 2.13)
More than high school vs. High school or less	1.29	(1.04, 1.62)

Odds ratios are only calculated for significant characteristics in Table 1.
